# Non-covalent organocatalyzed enantioselective cyclization reactions of α,β-unsaturated imines

**DOI:** 10.3762/bjoc.20.268

**Published:** 2024-12-10

**Authors:** Sergio Torres-Oya, Mercedes Zurro

**Affiliations:** 1 Departamento de Química Orgánica y Química Inorgánica, Instituto de Investigación Química “Andrés M. del Río” (IQAR), Universidad de Alcalá (IRYCIS), 28805 Madrid, Spain

**Keywords:** α,β-unsaturated imines, asymmetric organocatalysis, cyclization, *N*-heterocycles, inverse electron demand aza-Diels–Alder reaction

## Abstract

Asymmetric cycloaddition is a straightforward strategy which enables the synthesis of structurally distinct cyclic derivatives which are difficult to access by other methodologies, using an efficient and atom-economical path from simple precursors. In recent years several asymmetric catalytic cyclization strategies have been accomplished for the construction of *N*-heterocycles using various catalytic systems such as chiral metal catalysts, chiral Lewis acids or chiral organocatalysts. This review presents an overview of the recent advances in enantioselective cyclization reactions of 1-azadienes catalyzed by non-covalent organocatalysts.

## Introduction

Nitrogen-containing heterocycles are abundant scaffolds present in natural products, biologically active compounds, pharmaceuticals, synthetic agrochemicals, and functional materials [1–2]. Due to their importance, different synthetic routes involving stoichiometric and catalytic approaches have been developed.

The α,β-unsaturated imines, also known as conjugated imines or 1-azadienes, are useful precursors for the construction of aza-heterocycles. Due to their structure, they can be attacked by a nucleophile and undergo a 1,2-addition or conjugate addition leading to the production of allylic amines or aliphatic imines, respectively. They can also behave as C4 synthons in cycloaddition reactions such as the aza-Diels–Alder reaction, giving access to nitrogen-containing cyclic derivatives. Conjugated imines are usually synthesized from the corresponding carbonyl precursors by reaction with a sulfonamide in the presence of Lewis acids and a dehydrating agent such as molecular sieves [3]. Also, recently a palladium-catalyzed dehydrogenation of aliphatic imines was reported, providing a novel methodology for the construction of α,β-unsaturated imines [4].

**Figure 1:**
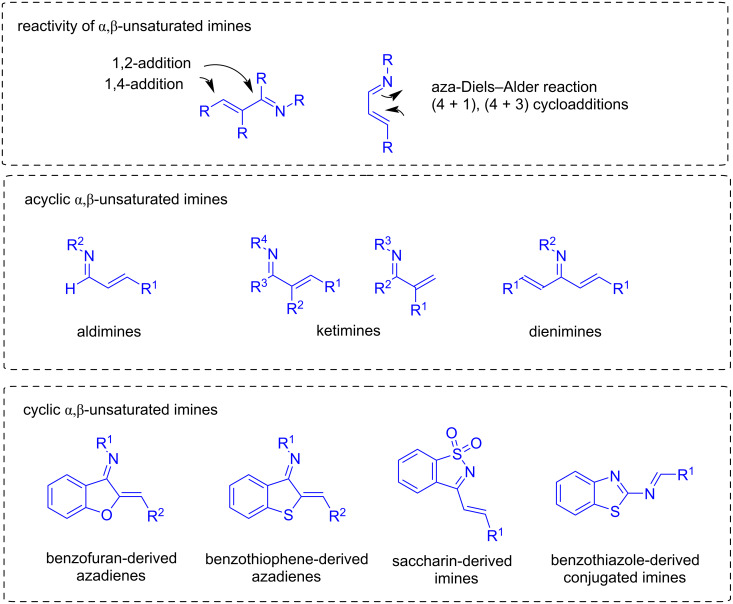
Reactivity of α,β-unsaturated imines and variety of structures.

There are different types of α,β-unsaturated imines, such as the acyclic imines aldimines, ketimines, or dienimines, depending on whether they are derived from aldehydes, ketones, or doubly unsaturated ketones, respectively. Additionally, the most common cyclic α,β-unsaturated imines involve benzofuran or saccharin-derived azadienes (Figure 1).

**Figure 2:**
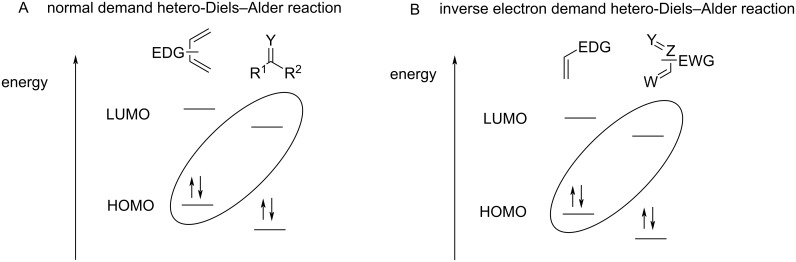
The hetero-Diels–Alder and inverse electron demand hetero-Diels–Alder reactions.

Cycloaddition reactions, especially Diels–Alder reactions, have attracted a lot of attention since their discovery as one of the most powerful methodologies for the construction of carbon–carbon bonds [5–10]. The hetero-Diels–Alder reaction is therefore an attractive strategy for the synthesis of heterocyclic compounds. It involves the reaction of dienes or dienophiles which possess a heteroatom in their structure. In this reaction, the HOMO of the diene and the LUMO of the dienophile interact to construct the six-membered heterocyclic derivative and the reaction requires electron-rich dienes and electron-poor dienophiles (Figure 2a). In the inverse electron demand hetero-Diels–Alder reaction (IEDHDA), the LUMO of the diene interacts with the HOMO of the dienophile, and therefore it proceeds through the reaction of electron-poor dienes and electron-rich dienophiles (Figure 2b).

**Figure 3:**
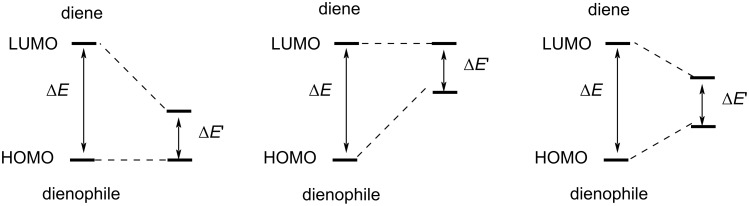
Different strategies to promote the activation of dienes and dienophiles in IEDADA reactions.

α,β-Unsaturated imines can undergo inverse electron demand aza-Diels–Alder reactions (IEDADA) to produce *N-*heterocyclic compounds. The search for an enantioselective pathway to carry out IEDADA reactions has been a glowing field in recent years [11–12]. In particular, organocatalysis can provide different activation modes to promote enantioselective IEDADA reactions [13–14], based on three strategies (Figure 3): i) LUMO-lowering activation (Brønsted acid catalysis), ii) HOMO-raising activation (amine-based catalysis and *N*-heterocyclic carbenes), and iii) LUMO-lowering and HOMO-raising activation (bifunctional thioureas and squaramides).

**Figure 4:**
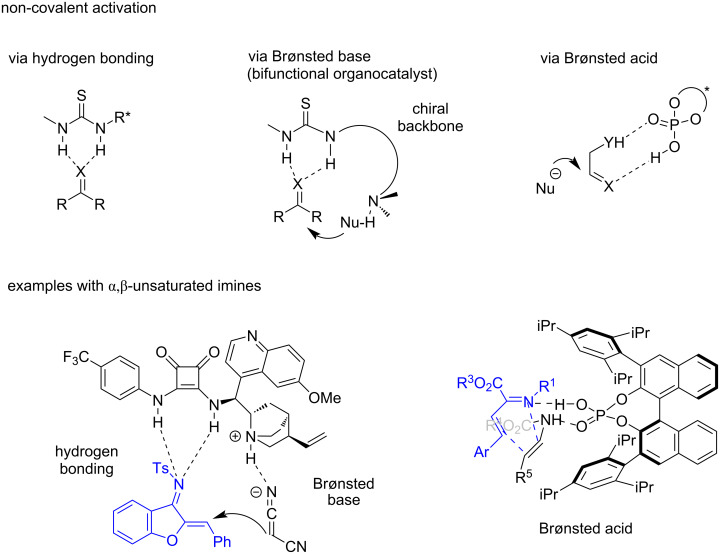
Examples of non-covalent interactions in organocatalysis.

Due to the ubiquitous nature of non-covalent interactions in organic systems, they can play a decisive role in asymmetric transformations [15]. Although quite important in all organocatalytic processes, there are specific organocatalysts which activate reactants through non-covalent interactions such as hydrogen bonding. These interactions are crucial to obtain high enantioselectivity in the reaction. The 1-azadienes possess an electronegative nitrogen atom which is prone to interacting with hydrogen-bond donors or Brønsted acids decreasing the LUMO energy of the diene. This review article aims to give an overview of the non-covalent organocatalyzed cyclization reactions involving α,β-unsaturated imines. Although most of the cyclization methodologies of 1-azadienes involve a formal [4 + 2] cycloaddition (IEDADA reaction) to construct six-membered nitrogen-containing molecules, α,β-unsaturated imines can also behave as C4 synthons involved in (4 + 1) and (4 + 3) cycloadditions or they act as C2 synthons undergoing (2 + 3) cycloadditions for example. This review discusses different examples involving IEDADA reactions and other cyclizations, with a special focus on the mode of action of the organocatalysts, and aims to show the synthetic applicability of the formed cyclic derivatives. The three non-covalent organocatalysts which will be covered in this review are hydrogen-bond donors such as thioureas and squaramides, Brønsted bases such as tertiary amines, and Brønsted acids such as chiral phosphoric acids. As depicted in Figure 4, a bifunctional squaramide is able to activate both an α,β-unsaturated imine through hydrogen bonding with the squaramide moiety and a nucleophile through deprotonation as Brønsted base. On the other hand, a chiral phosphoric acid provides a confined chiral environment where the reactants are approached, activating both the azadiene by interaction with the acidic hydrogen and a dienophile bearing a carbamate group by interaction with the oxygen atom of the phosphoryl group.

Although recently Rana and co-workers published a review article covering catalytic asymmetric transformations of azadienes [16], there is still room for this review which only focuses on non-covalent organocatalyzed cyclizations, and it will be a useful reference for organic chemists working in the field of asymmetric organocatalysis. The review is divided into sections, each covering a different catalytic system. Additionally, a chronological order is followed in the subchapters. In order to also give a general overview of the field, other dienes bearing two nitrogen atoms in their structure such as 1,3-azadienes or azo-alkenes are also included. On the contrary, asymmetric cyclizations involving aza-*ortho*-quinone intermediates and in situ-formed 1-azadienes were excluded as they have been discussed in other recent reviews [17–18].

## Review

### Hydrogen bond donors: bifunctional thioureas and squaramides

The use of bifunctional catalysts is commonplace in organocatalyzed transformations [19–23]. These catalysts are able to activate an electrophile and a nucleophile simultaneously and in IEDADA reactions they are employed to promote HOMO-raising and LUMO-lowering of both reactants leading to an enantioselective transformation. In this section, cyclization reactions of α,β-unsaturated imines catalyzed by hydrogen-bond donors, including bifunctional thioureas and squaramides bearing a Brønsted base moiety in their structure will be described.

**Scheme 1:**
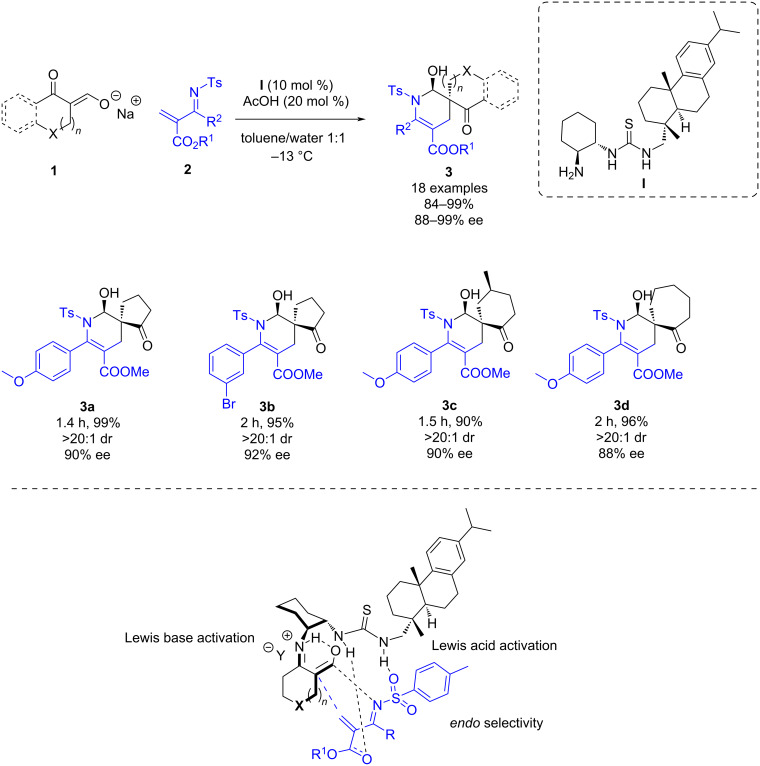
Enantioselective bifunctional thiourea-catalyzed inverse electron demand Diels–Alder reaction of *N*-tosyl-2-methylene-but-3-enoates and cyclic keto/enolate salts.

In 2012, Wang and co-workers reported a bifunctional thiourea-catalyzed aza-Diels–Alder reaction of cyclic keto/enolate salts **1** and *N*-tosyl-2-methylene-but-3-enoates **2** (Scheme 1). After a screening of the reaction conditions they found that organocatalyst **I**, acetic acid as additive and a mixture of toluene and water provided the best results in terms of yield and enantioselectivity. A wide scope was explored, including electron-donating substituents and electron-withdrawing groups, as well as heterocycles, giving densely functionalized chiral azaspirocyclic derivatives **3** in yields up to 99%, up to 20:1 dr, and up to 99% ee [24]. This work represents the first enantioselective bifunctional catalytic inverse electron demand Diels–Alder reaction that occurs with a dual control of the dienophile HOMO and diene LUMO energies of the substrates. The amino group of the organocatalyst acts as a Lewis base forming an enamine which raises the HOMO energy of the dienophile, while the thiourea moiety acts as a Lewis acid, lowering the LUMO level of the diene (Scheme 1). A confined transition state is formed providing a high enantiocontrol of the reaction.

**Scheme 2:**
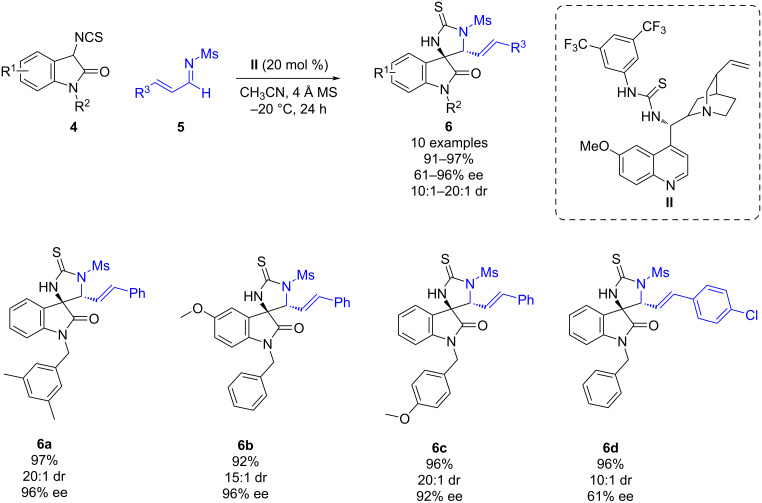
Cinchona-derived thiourea-catalyzed stereoselective (3 + 2) reaction of α,β-unsaturated imines and 3-isothiocyanatooxindole.

In 2016, Shi and co-workers reported a cinchona alkaloid-derived thiourea-catalyzed regio- and stereoselective cycloaddition of 3-isothiocyanatooxindoles and imines containing two or three electron-deficient unsaturated bonds [25]. Firstly, the (3 + 2) cycloaddition of 3-isothiocyanatooxindoles **4** and aldimines **5** was explored leading to the synthesis of spirooxindole derivatives **6** bearing a thiourea moiety in high yields (91–97%), and with good to excellent diastereoselectivities (10:1–20:1 dr) and enantioselectivities (61–96%) (Scheme 2).

**Scheme 3:**
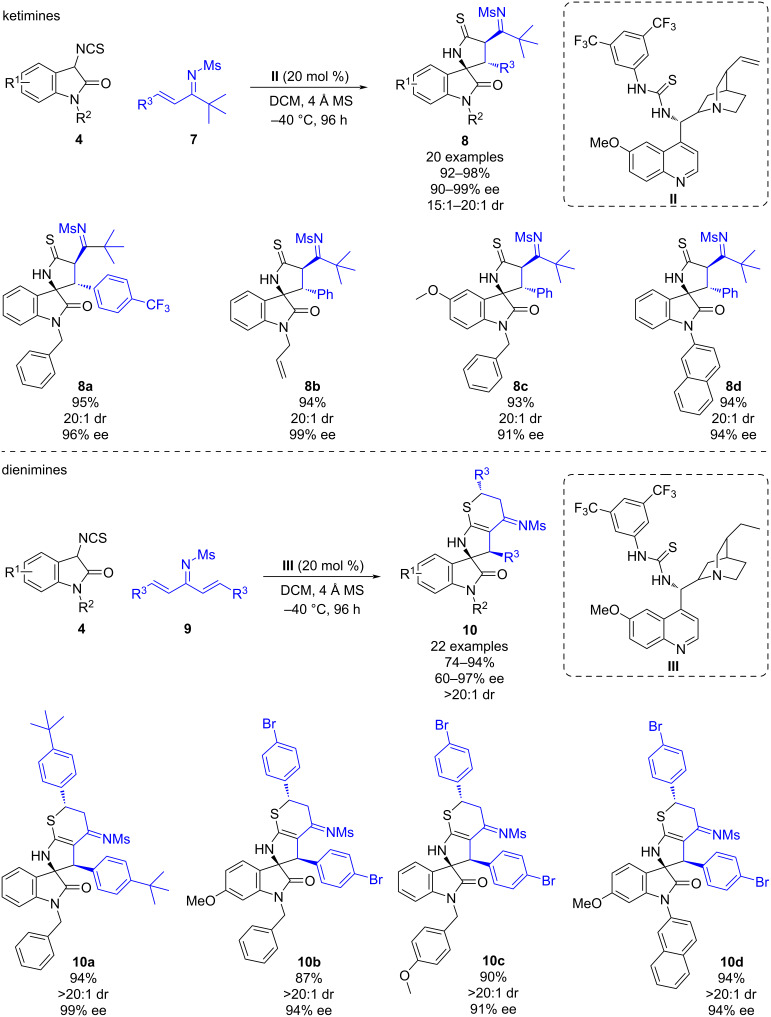
Cinchona-derived thiourea-catalyzed stereoselective (3 + 2)/(4 + 2) cascade reaction of α,β-unsaturated imines and 3-isothiocyanatooxindole.

Furthermore, the authors also investigated the reactivity of ketimines and dienimines (Scheme 3). The reaction of 3-isothiocyanatooxindoles **4** and ketimines **7** led to the (3 + 2) cycloaddition through the C=C bond of the α,β-unsaturated imine instead of the C=N bond, affording various spirocyclic derivatives **8** with excellent yields (92–98%), diastereoselectivities (15:1–20:1 dr), and enantioselectivities (90–99% ee). This result could be attributed to the higher steric hindrance at the carbon atom of the imine. On the other hand, the reaction of 3-isothiocyanatooxindoles **4** and dienimines **9** afforded the cascade cycloadducts **10** in high yields (74–94%) and excellent diastereoselectivities (>20:1 dr) and good to excellent enantioselectivities (60–97% ee) when using organocatalyst **III**.

**Scheme 4:**
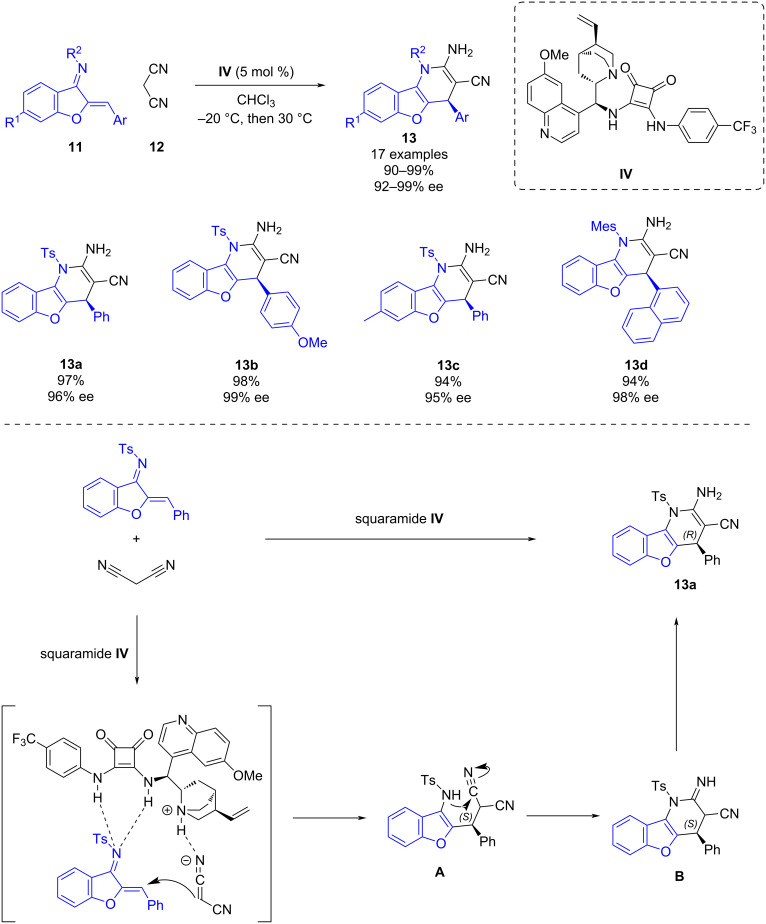
Enantioselective bifunctional squaramide-catalyzed formal [4 + 2] cycloaddition of malononitrile with benzofuran-derived azadienes.

Later, in 2018, Zhou and co-workers developed a bifunctional squaramide-catalyzed enantioselective formal [4 + 2] cycloaddition of benzofuran-derived azadienes **11** with malononitrile (**12**) [26]. This work provides an efficient methodology for synthesizing chiral benzofuran-fused 1,4-dihydropyridines **13** in excellent yields (90–99%) and excellent enantioselectivities (92–99% ee) (Scheme 4). The authors also attempted to perform the reaction using 2-tosylacetonitrile instead of malononitrile. However, in this case, the Michael addition product was obtained with low diastereoselectivity.

The mechanism is described in Scheme 4: the bifunctional squaramide activates the azadiene through hydrogen bonding while the malononitrile is deprotonated by the tertiary amine present in the backbone of the catalyst, establishing hydrogen bonding with the protonated tertiary amine. Then, a Michael addition of malononitrile to the azadiene takes place to obtain exclusively the (*S*)-intermediate **A**. Subsequently an intramolecular nucleophilic addition of the nitrile leads to the intermediate **B**, which undergoes tautomerization to furnish the cycloaddition product **13a**.

**Scheme 5:**
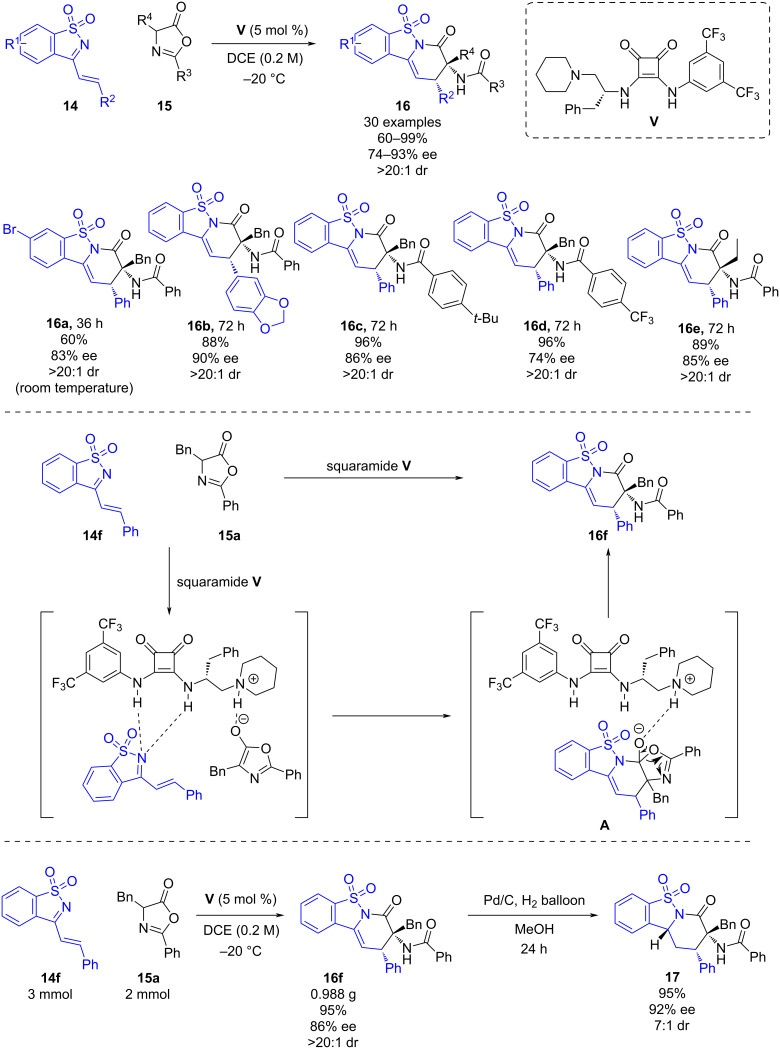
Bifunctional squaramide-catalyzed IEDADA reaction of saccharin-derived 1-azadienes and azlactones.

In 2019, Xu’s group published a bifunctional squaramide-catalyzed inverse electron demand aza-Diels–Alder reaction of saccharin-derived 1-azadienes **14** and azlactones **15** [27]. This methodology enables chiral tricyclic derivatives **16** bearing a quaternary amino acid moiety in up to 99% yields, up to 93% ee and >20:1 dr (Scheme 5) to be obtained. In general, the steric and electronic properties of the conjugated imines had a slight effect on the enantioselectivities of the reactions. However, the authors pointed out that the azlactones with different substituents at the α*-*position led to the desired products albeit with unsatisfactory results.

The bifunctional squaramide catalyst **V** has two functions; firstly it deprotonates the enolic form of the azlactone through the Brønsted-base moiety, and secondly it activates the 1-azadiene and enolate form of the azlactones through H-bond interactions with the squaramide moiety. The activated complex undergoes a [4 + 2] cyclization, through the *Si*-face attack of the enolate to the 1-azadiene leading to intermediate **A** which undergoes tautomerization and protonation to yield the chiral tricyclic derivative **16**.

To further investigate the potential utility of this methodology, a gram scale experiment was conducted affording product **16f** in a good yield and a slight decrease of the enantioselectivity. Additionally, a derivatization of product **16f** by hydrogenation was carried out to yield the tricyclic piperidine **17** with high diastereoselectivity (Scheme 5).

**Scheme 6:**
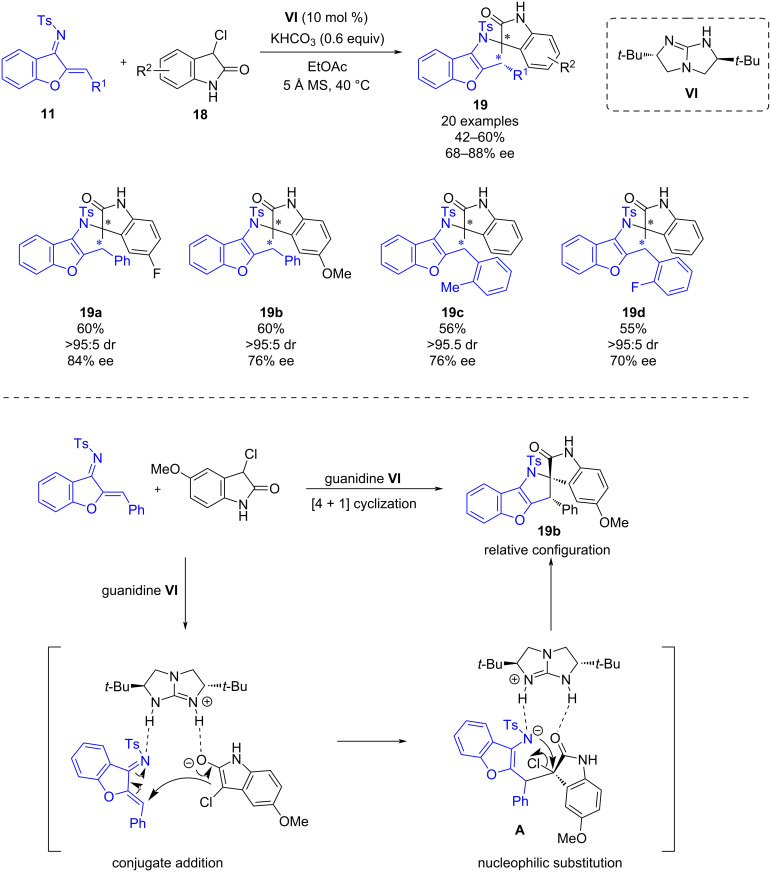
Chiral guanidine-catalyzed enantioselective (4+1) cyclization of benzofuran-derived azadienes with 3-chlorooxindoles.

In 2019, Shi and co-workers established a guanidine-catalyzed enantioselective (4 + 1) cyclization of benzofuran-derived azadienes **11** with 3-chlorooxindoles **18** [28]. This work provides a useful strategy for the synthesis of chiral spirooxindole derivatives **19** in moderate yields (42–60%), high diastereoselectivities and good enantioselectivities (68–88% ee) (Scheme 6). The authors also attempted the reaction using *N*-benzyl-protected 3-chlorooxindole as a substrate. However, in this case, no reaction was observed, which indicated that the N–H group of the 3-chlorooxindole **18** has an essential role in the reaction.

The proposed reaction pathway for the enantioselective (4 + 1) cyclization is illustrated in Scheme 6. Initially, chiral guanidine **VI**, acts as a Brønsted base deprotonating the enolic form of 3-chlorooxindole whilst simultaneously activating the benzofuran-derived azadiene by H-bonding. This dual activation promotes a stereoselective addition of 3-chlorooxindole to the azadiene leading to intermediate **A**. The latter is also activated by the chiral guanidine and undergoes an intramolecular nucleophilic substitution which delivers the product **19b** with the relative configuration depicted in Scheme 6.

**Scheme 7:**
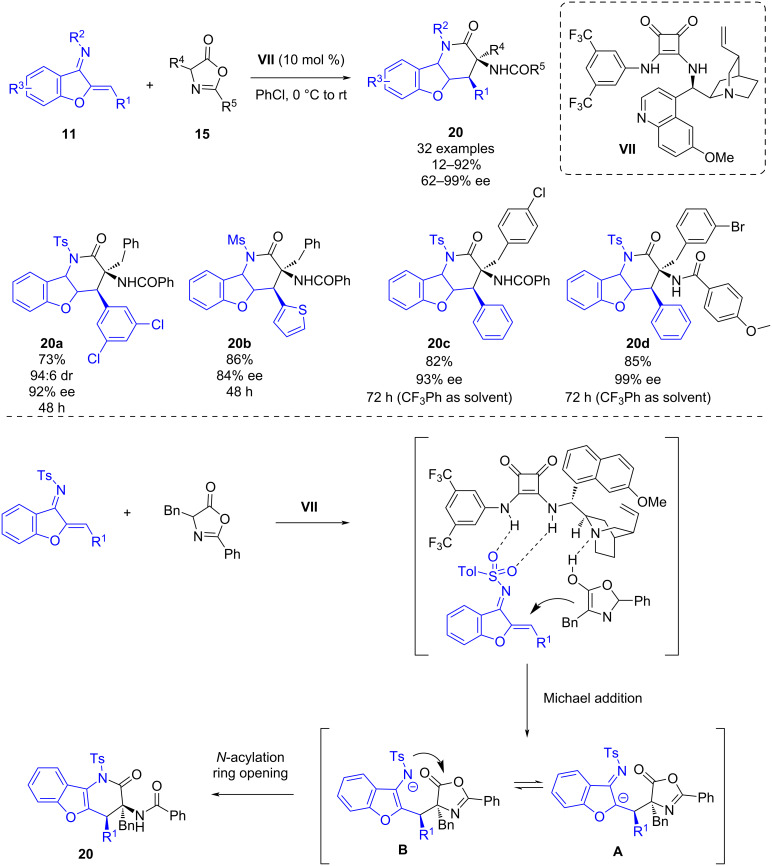
Bifunctional squaramide-catalyzed [4 + 2] cyclization of benzofuran-derived azadienes and azlactones.

Also in 2019, Huang and co-workers reported a bifunctional squaramide-catalyzed [4 + 2] cyclization of benzofuran-derived azadienes **11** and azlactones **15** [29]. This methodology enables the synthesis of benzofuran-fused six-membered heterocycles **20** in yields up to 92%, complete diastereoselectivities and moderate to excellent enantioselectivities (62–99% ee) (Scheme 7). The 1-azadienes bearing electron-rich or electron-poor groups on the *meta* or *para*-position of the phenyl ring led to the derivatives **20** with excellent enantioselectivities. However, when 1-azadienes bearing substituents at the *ortho*-position were tested, the enantioselectivities decreased, probably due to higher steric hindrance.

The mechanism of the reaction is depicted in Scheme 7; firstly, the dual activation of the azadiene and the enol form of the azlactone through hydrogen bonding with the bifunctional squaramide enables a stereoselective Michael addition of the azlactone to the azadiene forming intermediate **A** which transforms into intermediate **B**, which then undergoes an intramolecular cyclization to give the desired product **20**.

**Scheme 8:**
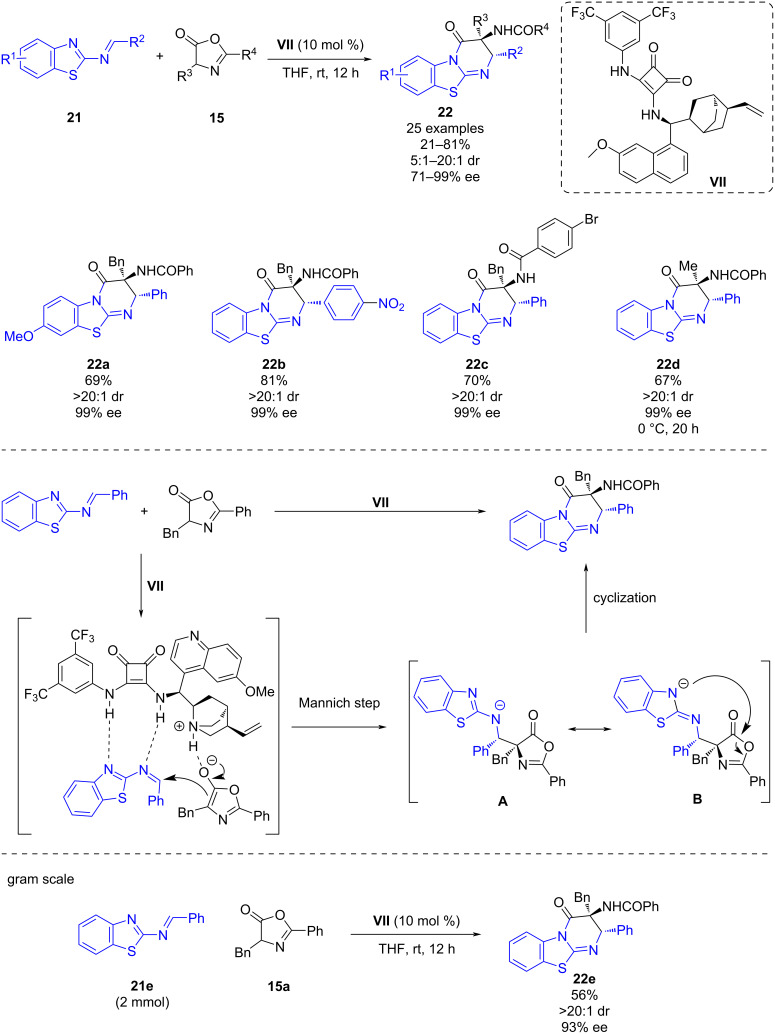
Chiral bifunctional squaramide-catalyzed domino Mannich/formal [4 + 2] cyclization of 2-benzothiazolimines with azlactones.

In 2020, Ni, Song and co-workers described a bifunctional squaramide-catalyzed asymmetric domino Mannich/formal [4 + 2] cyclization of 2-benzothiazolimines **21** with azlactones **15** [30]. Using cinchona-derived squaramide **VII** it was possible to obtain 25 different chiral benzothiazolopyrimidines **22** bearing adjacent tertiary and quaternary stereocenters in up to 81% yield, up to 20:1 dr, and up to 99% ee (Scheme 8).

The organocatalyst has a bifunctional role; firstly, the tertiary amine deprotonates the azlactone providing its enolate form while at the same time the squaramide moiety activates the 2-benzothiazolimine by hydrogen-bonding interactions with the nitrogen atoms. Then, the azlactone enolate undergoes a nucleophilic attack on its *Si*-face via Mannich reaction with the 2-benzothiazolimine leading to intermediate **A** which evolves toward its resonance form **B**, responsible of the intramolecular attack of the negatively charged nitrogen to the azlactone moiety leading to the desired chiral benzothiazolopyrimidine. Furthermore, a large-scale experiment was carried out obtaining the product **22e** in 56% yield, >20:1 dr and 93% ee (Scheme 8).

**Scheme 9:**
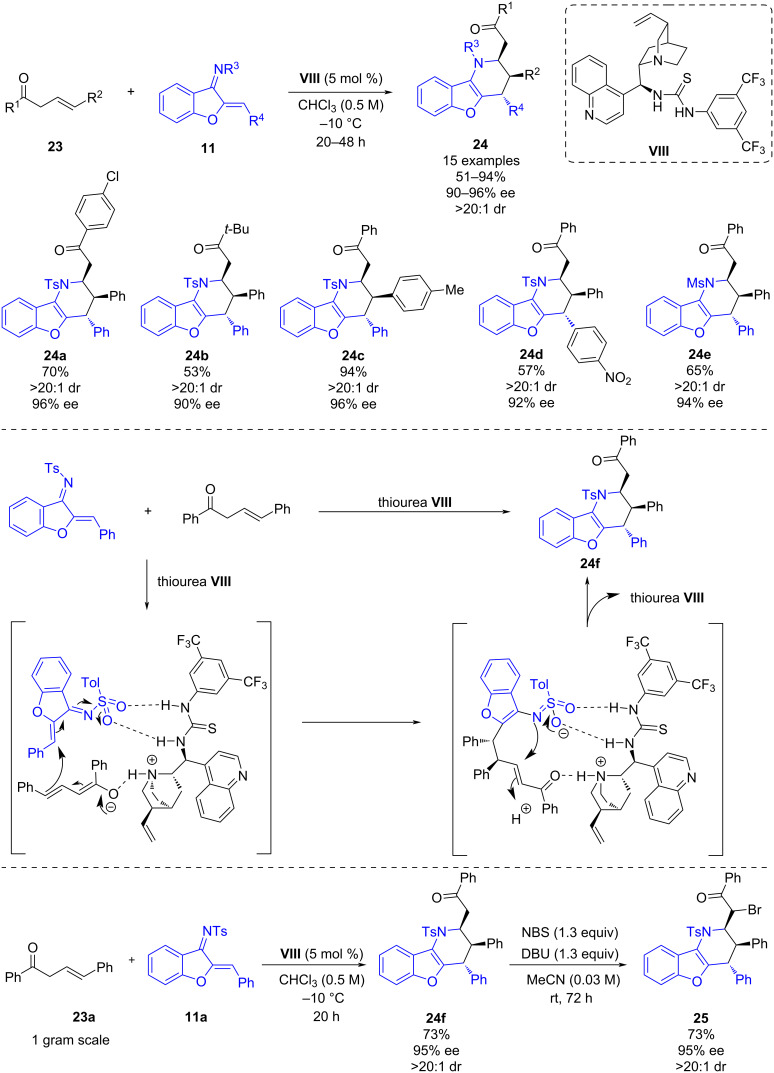
Chiral bifunctional thiourea-catalyzed formal IEDADA reaction of β,γ-unsaturated ketones and benzofuran-derived azadienes.

In May 2020, Albrecht and co-workers reported a chiral bifunctional thiourea-catalyzed formal inverse electron demand aza-Diels–Alder reaction (IEDADA) of β,γ-unsaturated ketones **23** and benzofuran-derived azadienes **11** [31]. The procedure enables the straightforward synthesis of benzofuran derivatives **24** bearing an additional tetrahydropyridine ring in good yields (51–94%) and excellent enantioselectivities (90–96% ee) when using thiourea **VIII** with a relatively low catalyst loading (Scheme 9). The authors also attempted to perform the reaction using acyclic azadienes and indene-derived azadiene instead of benzofuran-derived azadienes **11**. However, in these cases, the reactions did not take place.

The authors proposed that the bifunctional thiourea catalyst acts by activating the sulfonyl group of the imine by hydrogen-bonding interactions while at the same time deprotonating the β,γ-unsaturated ketone forming the corresponding dienolate. The latter then participates in the formal IEDADA reaction with the azadiene in a stepwise mechanism: firstly, the vinylogous Michael addition of the dienolate to the double bond of the α,β-unsaturated *N*-sulfonylimine occurs in a stereoselective fashion. Subsequently, cyclization due to an intramolecular aza-Michael reaction and protonation leads to the enantioenriched product **24f**. To show the applicability of this novel methodology, a gram-scale reaction was carried out obtaining comparable results as in a small scale. Besides, an α-bromination of the product **24f** was performed obtaining **25** in 73% yield and complete diastereoselectivity (Scheme 9).

**Scheme 10:**
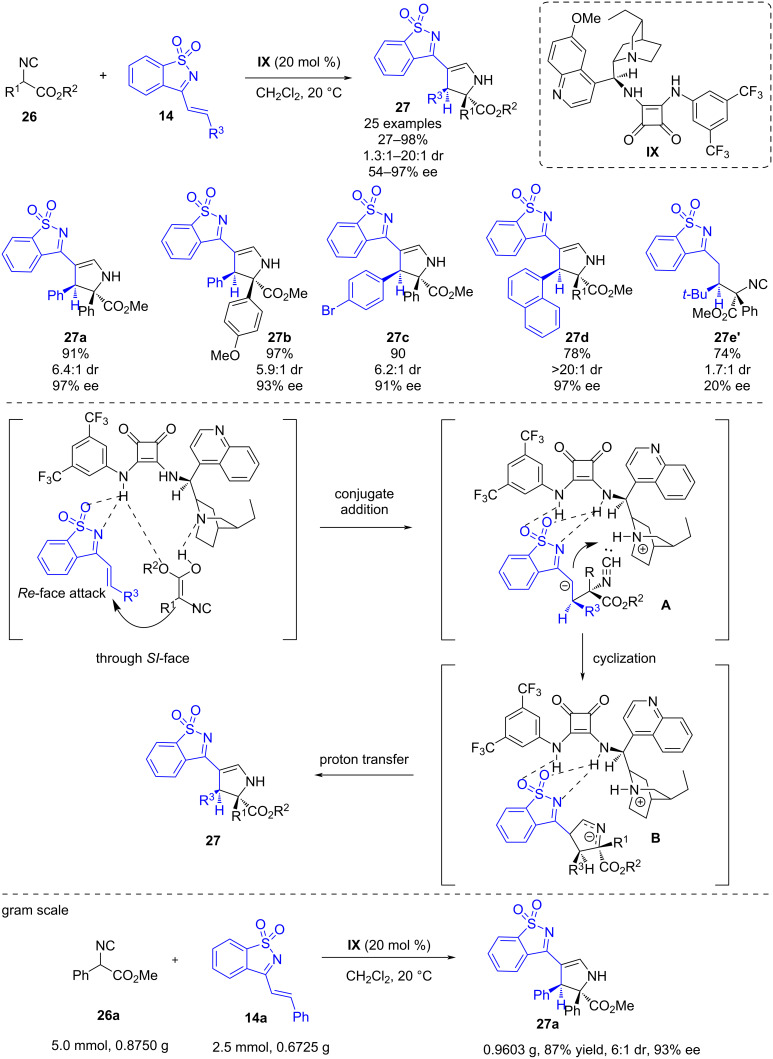
Dihydroquinine-derived squaramide-catalyzed (3 + 2) cycloaddition reaction of isocyanoacetates and saccharin-derived 1-azadienes.

In 2021, Zhao and co-workers reported a dihydroquinine-derived squaramide-catalyzed (3 + 2) cycloaddition reaction of isocyanoacetates **26** and saccharin-derived 1-azadienes **14** [32]. In this work, the azadiene works as a C2 synthon, while the isocyanoacetate, bearing a protected carboxylic acid and a carbene-like divalent isocyanide carbon, behaves a C3 synthon affording benzo[*d*]isothiazole 1,1-dioxide-dihydropyrroles **27** bearing two adjacent stereocenters in high yields (27–98%) and with moderate to excellent stereoselectivities (54–97% ee) when using thiourea **IX** (Scheme 10). The cyclization was observed with all derivatives except for azadienes bearing a bulky *t*-Bu substituent, which led to the Michael product **27e’** albeit with low stereoselectivity. The authors proposed a plausible reaction mechanism to explain the observed stereoselectivity of the reaction. Firstly, the isocyanoacetate is deprotonated by the tertiary amine moiety of the organocatalyst, while the azadiene is activated by hydrogen bonding with the squaramide moiety. Then, the enolate attacks the *Re* face of the azadiene through a Michael addition which leads to intermediate **A**. Next, an intramolecular 5-*endo*-*dig* cyclization takes place by the attack of the carbanion to the isocyanate group forming intermediate **B** which undergoes protonation yielding the product. The authors attempted to perform different reductions of the products without any success. However, they performed a gram scale version of the transformation obtaining the product in comparable yields and enantioselectivities.

**Scheme 11:**
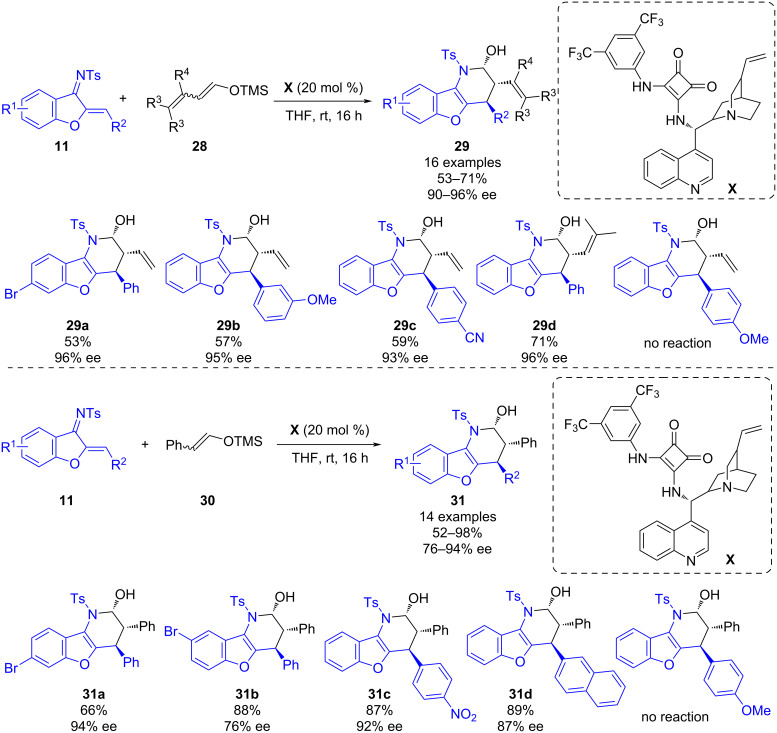
Enantioselective squaramide-catalyzed asymmetric IEDADA reaction of benzofuran-derived azadienes and silyl (di)enol ethers.

Later in 2021, Fernández-Salas, Alemán and co-workers developed a bifunctional squaramide-catalyzed enantioselective inverse electron demand aza-Diels–Alder reaction (IEDADA) between benzofuran-derived azadienes and silyl (di)enol ethers [33]. This work provides a useful methodology to synthesize interesting benzofuran-fused 2-piperidinol derivatives **29** and **31** bearing three adjacent stereocenters using squaramide **X** as organocatalyst (Scheme 11). Firstly, the reaction between azadiene **11** and trimethylsilyl dienolate **28** was explored obtaining 16 different derivatives in moderate to good yields (53–71%) and excellent enantioselectivities (90–96% ee). Furthermore, with the idea to expand the scope of the reaction, the authors studied the reaction using trimethyl(styryloxy)silane (**30**). This strategy enabled the synthesis of adducts **31** bearing an aromatic ring at the α-position of the activating group with good yields (52–98%), excellent diastereoselectivities and good enantioselectivities (76–94% ee). Additionally, to understand the mechanism of the reaction, the authors performed DFT calculations. Based on the calculations, once the dienolate is hydrolyzed to the free alcohol *E* isomer and it coordinated to the squaramide in an effective orientation, a stepwise mechanism is taking place. Firstly, the dienolate adds to the azadiene which is followed by cyclization with formation of the C–N bond. Finally, protonation leads to the formation of the desired derivative. The calculations also revealed that due to kinetic control the activation of the C2 carbon of the dienolate is preferred vs the C4 atom, therefore only one regioisomer is observed.

**Scheme 12:**
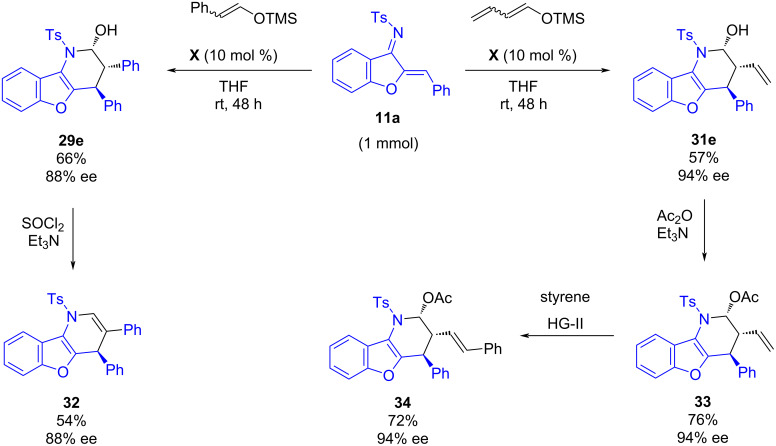
Scale up and derivatizations of benzofuran-fused 2-piperidinol derivatives.

Additionally, the asymmetric IEDADA reaction could be performed at a higher scale (1 mmol) leading to the synthesis of the cycloadducts **29e** and **31e** in good yields (Scheme 12). Further derivatizations were also carried out: The treatment of **29e** with SOCl_2_ led to interesting unsaturated derivative **32** in a 54% yield. The acetylation of **31e** provided **33** in 76% yield. Next, an alkene metathesis of **33** with styrene led to product **34** in 72% yield. All the derivatizations were carried out without erosion of enantioselectivity.

**Scheme 13:**
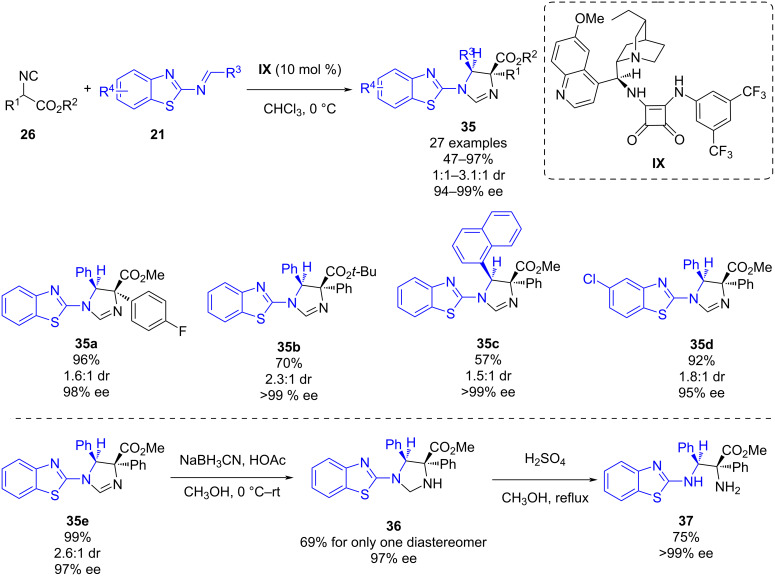
Dihydroquinine-derived squaramide-catalyzed Mannich-type reaction of isocyanoacetates with *N*-(2-benzothiazolyl)imines.

In 2023, as a follow-up of Zhao’s previous work [32], the same group reported a dihydroquinine-derived squaramide-catalyzed Mannich-type reaction of isocyanoacetates with *N*-(2-benzothiazolyl)imines [34]. After a screening of reaction conditions, it was found that the best organocatalyst was also bifunctional squaramide **IX**, but in this case a lower catalyst loading could be used in the reaction (Scheme 13). Under the optimized reaction conditions, 27 different chiral directly linked benzothiazole-dihydroimidazoles **35** bearing two adjacent stereocenters could be obtained in moderate to high yields (47–97%), moderate diastereoselectivities (1:1–3.1:1 dr) and excellent enantioselectivities (94–99% ee). It is worth noting that the steric effect of the isocyanate substituents had an important effect on the reaction, and with bulky substituents, the reaction did not proceed. Further derivatizations of the products were carried out: firstly, the derivative **35e** was reduced using NaBH_3_CN to obtain **36** as a single diastereomer in a good yield. Next, a hydrolysis of **36** using concentrated sulfuric acid led to the 1,2-diamino derivative **37** in a good yield and an excellent enantioselectivity.

### Brønsted bases

**Figure 5:**
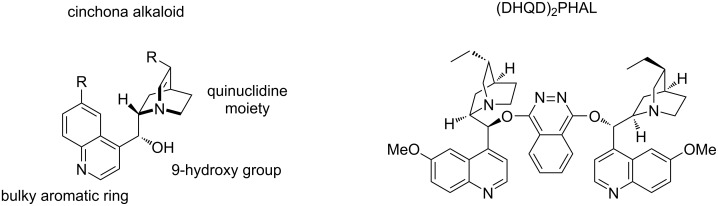
Structure of a cinchona alkaloid and (DHQD)_2_PHAL.

Cinchona alkaloids have been established as highly efficient chiral organocatalysts, capable to engage in a wide array of enantioselective processes [35]. They possess a unique structure. Firstly, the bulky quinuclidine moiety can act as a base activating a nucleophile, secondly the secondary hydroxy group can participate in hydrogen bonding or can behave as a Brønsted acid (Figure 5). Additionally, the quinoline moiety can interact through π–π stacking with the reactants.

On the other hand, the (DHQD)_2_-based catalysts are synthesized by joining together two fragments of cinchona alkaloids. In this manner, it is possible to obtain a symmetric catalyst, which can engage in hydrogen bonding interactions and deprotonation processes. Although they were originally used for Sharpless dihydroxylation, they have been utilized for a wide variety of organocatalytic processes.

In this section, different cyclizations of α,β-unsaturated imines involving Brønsted base organocatalysts such as (DHQD)_2_-based catalysts will be described.

**Scheme 14:**
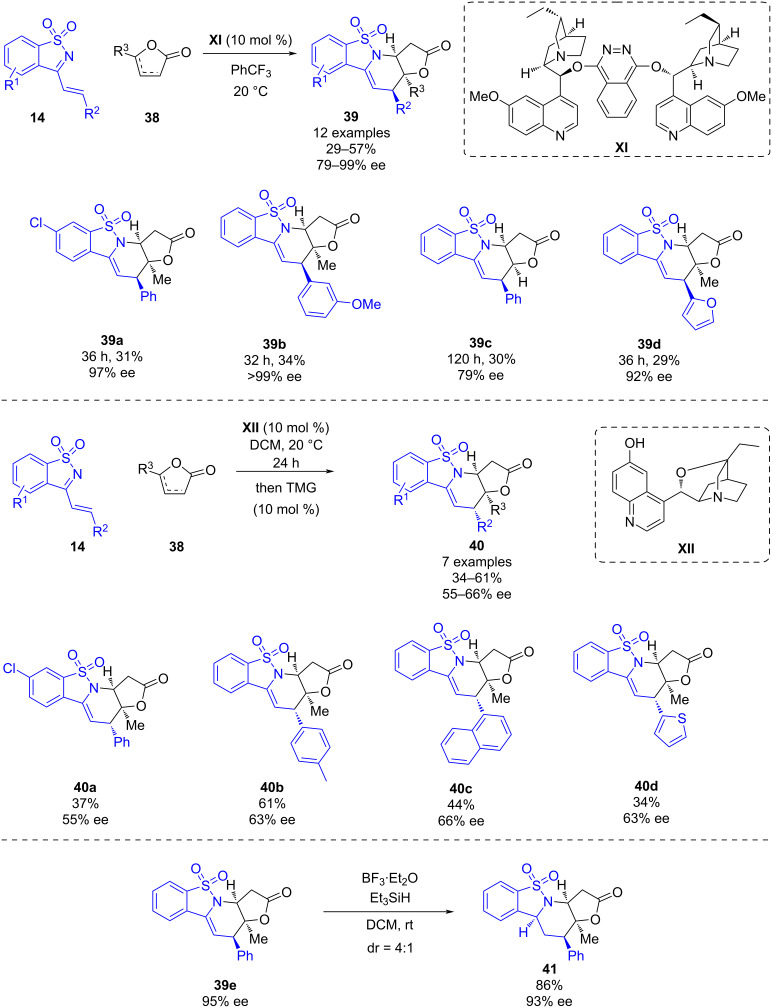
Enantioselective modified cinchona alkaloid-catalyzed [4 + 2] annulation of γ-butenolides and saccharin-derived 1-azadienes.

In 2015, Jiang, Chen and co-worker published a modified cinchona alkaloid-catalyzed [4 + 2] cycloaddition reaction of saccharin-derived 1-azadienes **14** and γ-butenolides **38** [36]. While using (DHQD)_2_PHAL **XI**, an *endo* cycloadduct **39** could be obtained in good yields (29–57%) and high enantioselectivity (79–99% ee), and the use of the β-isocupreidine-based catalyst **XII** as a catalyst led to the *exo*-type diastereomers **40** with moderate yields (34–61%) and moderate enantioselectivities (55–66% ee). In this case, the addition of tetramethylguanidine (TMG) was necessary to promote the cyclization. This work is one of the first reports in which an enantioselective [4 + 2] cycloaddition of 1-azadienes was performed under Brønsted base conditions. The authors claimed that the reactions to obtain the complex fused polycyclic diastereomeric products is proceeding through a cascade Michael addition–aza-Michael addition, however, they did not propose a plausible reaction pathway for the transformations. In order to evaluate the synthetic utility of this methodology, derivative **39e** was reduced using BF_3_·OEt_2_ and Et_3_SiH leading to **41** in a good yield and moderate diastereoselectivity (Scheme 14).

**Scheme 15:**
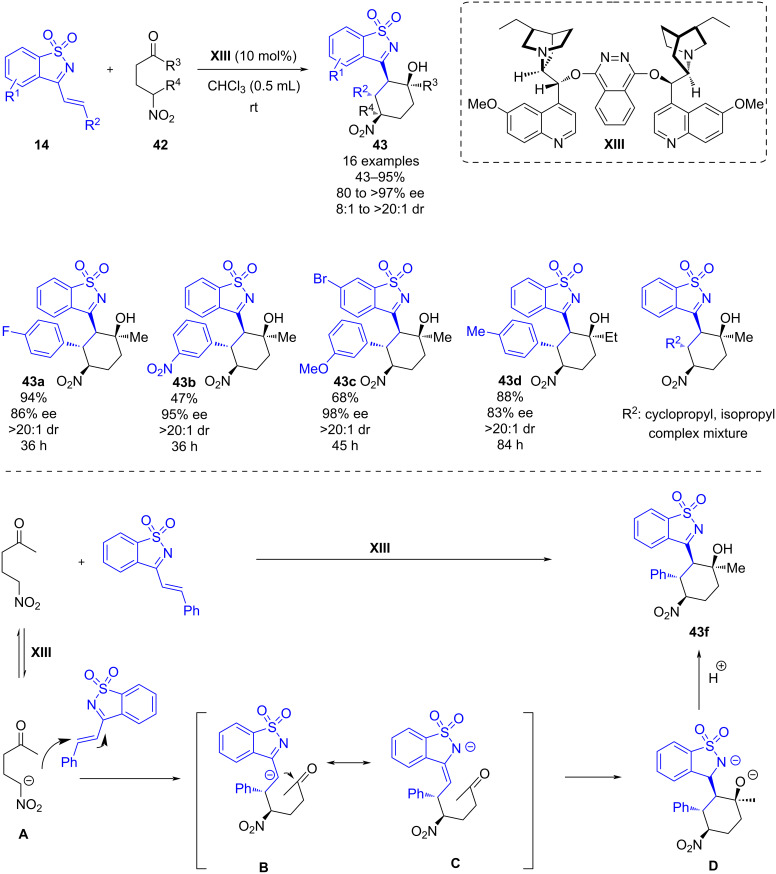
Chiral tertiary amine-catalyzed [2 + 4] annulation of cyclic 1-azadiene with γ-nitro ketones.

In 2017, Li’s group developed a chiral (DHQ)_2_PHAL-catalyzed [2 + 4] annulation reaction of cyclic 1-azadiene **14** with γ-nitro ketones **42** [37]. With this transformation polysubstituted cyclohexanes **43** bearing four consecutive stereocenters are afforded through efficient one-pot cyclization with good yields (43–95%) and with high enantioselectivities (80–97% ee) (Scheme 15). In general, 1-azadienes **14** with different substitution patterns and aryl R^2^ substituents led to the desired products. However, the presence of a cyclopropyl or isopropyl R^2^ substituent, led to a complex mixture of products (Scheme 15).

In the mechanism proposed by the authors, (DHQ)_2_PHAL **XIII** provokes the deprotonation of the γ-nitro ketone at the α-position to the nitro group, resulting in the formation of an anionic nucleophilic specie **A**. The latter reacts with the cyclic 1-azadiene via stereoselective Michael addition, forming intermediate **B**, stabilized by π–π stacking interactions between the Ar group and the aromatic cyclic moiety of the 1-azadiene. Then, an intramolecular aldol reaction leads to intermediate **D** which undergoes final protonation to produce the desired chiral product **43f**. The authors proposed that the stereoselectivity of the Michael addition product would direct the stereoselectivity of the intramolecular aldol reaction. To demonstrate the robustness of this novel methodology the model reaction was scaled up to 1.0 mmol, giving the desired product with a slight decrease of the yield and similar results in terms of enantioselectivity, 67% yield, 92% ee and >20:1 dr.

### Brønsted acids: chiral phosphoric acids

The employment of Brønsted acid catalysis has been widely studied in asymmetric synthesis [38–39]. While the asymmetric transformations of 2-azadienes have been more intensively investigated, enantioselective derivatizations of 1-azadienes are scarce. In this section, the cycloaddition reactions involving α,β-unsaturated imines catalyzed by chiral phosphoric acids will be described.

**Scheme 16:**
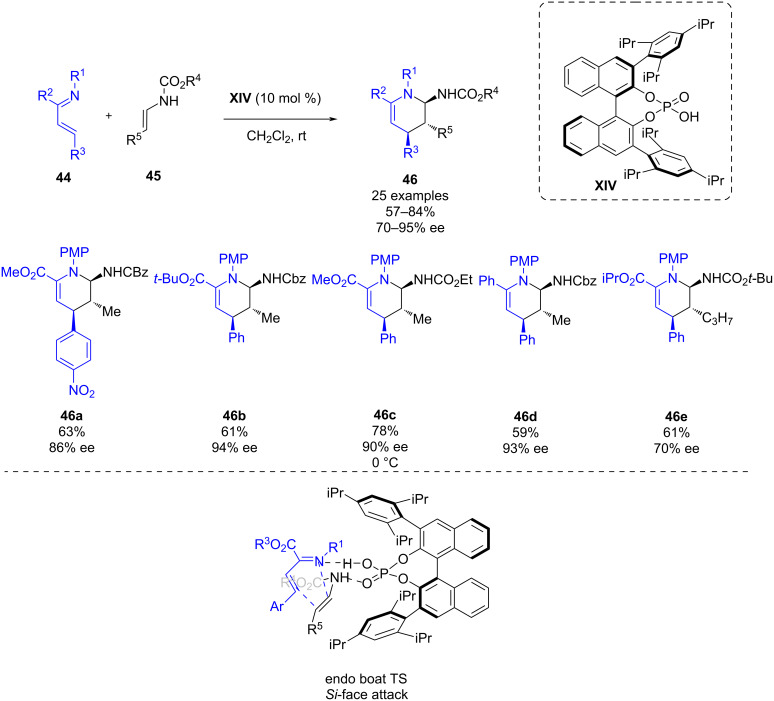
Inverse electron demand aza-Diels–Alder reaction (IEDADA) of 1-azadienes with enecarbamates catalyzed by chiral phosphoric acid **XIV**.

In 2013, Masson and co-workers reported the enantioselective inverse electron demand aza-Diels–Alder reaction (IEDADA) of 1-azadienes **44** with enecarbamates **45** catalyzed by chiral phosphoric acid **XIV** [40]. Although other works involving a covalent attachment of the catalyst had been previously reported for IEDADA reactions, this work reflects the first Brønsted acid-catalyzed IEDADA reaction of 1-azadienes. This methodology enables the synthesis of a variety of chiral tetrahydropyridine derivatives **46** in good yields (57–84%) and good to excellent enantioselectivities (70–95% ee) when using chiral phosphoric acid **XIV** (Scheme 16).

Mechanistic studies were performed to unveil whether a concerted or stepwise mechanism is taking place, such as trying to trap a possible iminium ion intermediate. The outcome of those experiments pointed towards a concerted mechanism which could be explained by the transition state depicted in Scheme 16: the chiral phosphoric acid could act as a bifunctional catalyst; the OH group acts as a Brønsted acid activating the 1-azadiene, while the phosphoryl oxygen atom activates the enecarbamates as a Lewis base. In order to explain the stereochemistry of the product of the IEDADA reaction, the *Si* face of the azadiene should be attacked by the enecarbamate with *endo* selectivity, thus leading to the (4*S*,5*R*,6*R*)-cycloadduct **46**.

**Scheme 17:**
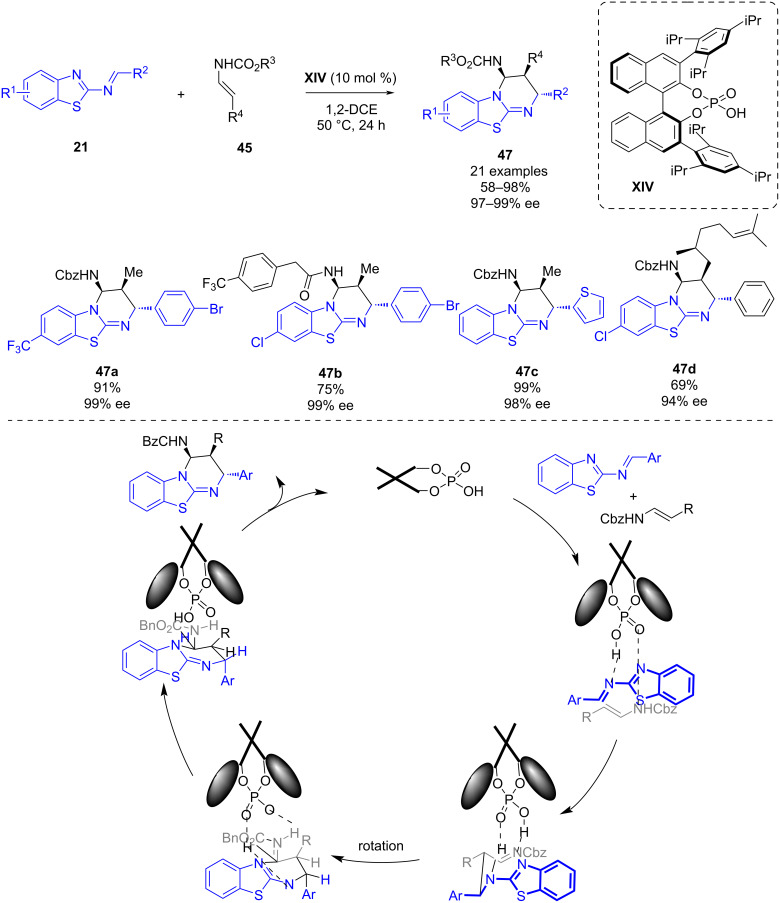
Phosphoric acid-catalyzed enantioselective [4 + 2] cycloaddition of benzothiazolimines and enecarbamates.

In 2019, as a follow-up work of their previous work, Masson and co-workers published a phosphoric acid-catalyzed enantioselective [4 + 2] cycloaddition of benzothiazolimines **21** and enecarbamates **45** [41]. With this novel methodology benzothiazolopyrimidines **47** bearing three contiguous stereogenic centers were synthesized in good to excellent yields (58–98%), complete diastereoselectivities and excellent enantioselectivities (97–99% ee) using also chiral phosphoric acid **XIV** (Scheme 17). The authors proposed a reaction mechanism in order to explain the observed stereoselectivity of the products in which through hydrogen bonding the chiral phosphoric acid provides a chiral environment where the reaction takes place (Scheme 17).

**Scheme 18:**
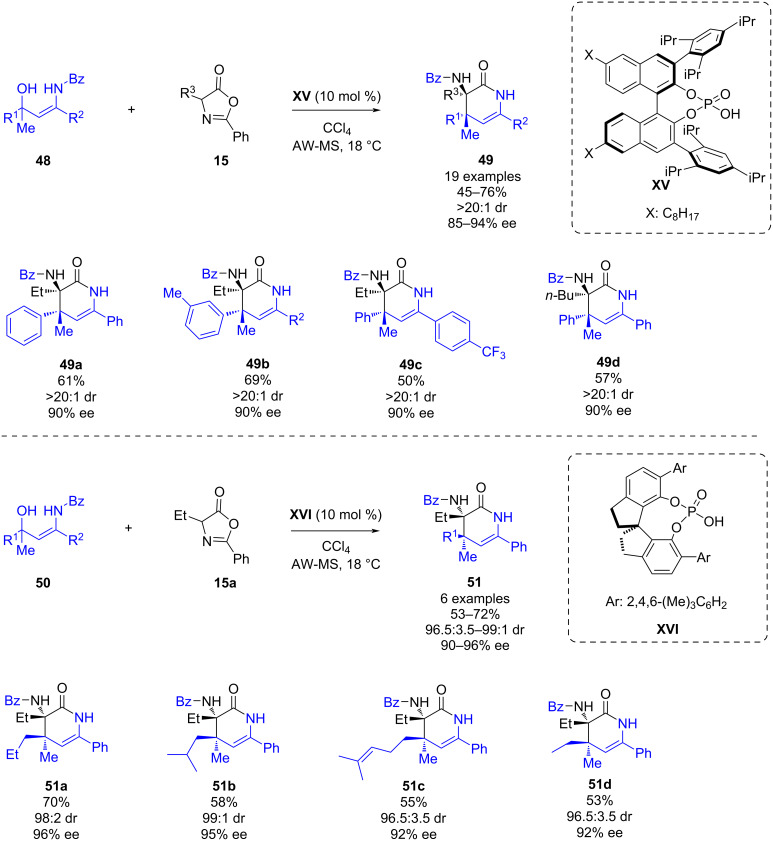
Phosphoric acid-catalyzed enantioselective inverse electron demand aza-Diels–Alder reaction of in situ-generated β,β-disubstituted α,β-unsaturated N–H ketimines and azlactones.

In 2020, He, Yang and co-workers reported a phosphoric acid-catalyzed enantioselective inverse electron demand aza-Diels–Alder reaction of in situ-generated β,β-disubstituted α,β-unsaturated N–H ketimines and azlactones [42]. This methodology enabled the synthesis of dihydropyridinones **49** and **51** in moderate to good yields (45–76%), excellent diastereoselectivities and high enantioselectivities (85–94% ee) (Scheme 18). The reaction was also compatible with 1,1-dialkyl-substituted 3-amidoallylic alcohols **50** as precursors of α,β-unsaturated N–H ketimines; in this case the chiral SPINOL-derived phosphoric acid **XVI** provided the best enantioselectivities (Scheme 18). In both reactions, it was necessary to add acid-washed molecular sieves (AW-MS) in the reaction medium.

**Scheme 19:**
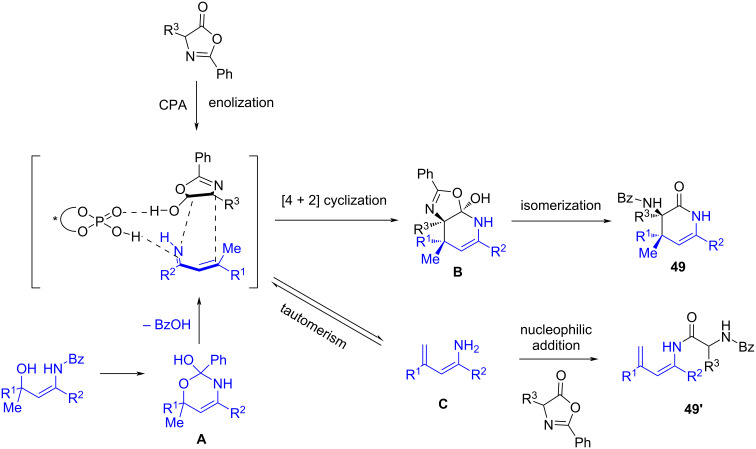
Proposed reaction mechanism for the phosphoric acid-catalyzed enantioselective inverse electron demand aza-Diels–Alder reaction of in situ generated β,β-disubstituted α,β-unsaturated N–H ketimines and azlactones.

The proposed reaction mechanism based on the mechanistic experiments and previous reports is depicted in Scheme 19: the azlactone is activated by the chiral phosphoric acid to generate its active enol form, while at the same time the chiral phosphoric acid mediates the in situ formation of the α,β-unsaturated N–H ketimine, occurring through the formation of an orthoester intermediate **A**. Then, the chiral phosphoric acid activates both the enol form of the azlactone and the α,β-unsaturated imine, facilitating the asymmetric [4 + 2] cyclization reaction. The authors proposed the formation of intermediate **B** which subsequently undergoes an isomerization to give the chiral dihydropyridinone derivative **49**. An alternative pathway which occurs is the transformation of the α,β-unsaturated imine into the corresponding enamine **C**, which attacks to the azlactone leading to the addition subproduct **49’**, also observed in the catalytic reactions.

**Scheme 20:**
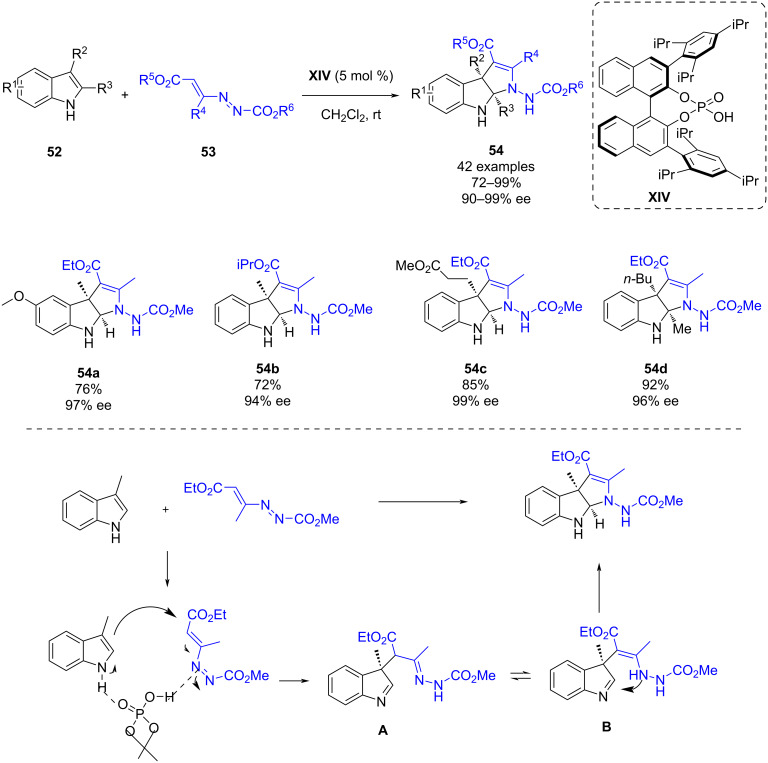
Enantioselective dearomatization of indoles by a (3 + 2) cyclization with azoalkenes catalyzed by a chiral phosphoric acid.

Also in 2020, Lu and co-workers reported an enantioselective dearomatization of indoles **52** via a (3 + 2) cyclization with azoalkenes **53** catalyzed by chiral phosphoric acid **XIV** (Scheme 20). This methodology enables the synthesis of a wide scope of pyrroloindolines **54**, important privileged polycyclic indolines in high yields (72–99%) and high enantioselectivities (90–99%) [43].

The authors proposed a possible mechanism for the reaction which is depicted in Scheme 20. The chiral phosphoric acid activates both the azoalkene acting as a Brønsted acid and the indole acting as a Lewis base promoting a Friedel–Crafts-type 1,4-addition of the indole to the azoalkene. In this manner, a hydrazone intermediate **A** is formed, which tautomerizes to the enamine **B**. Finally, this intermediate undergoes an intramolecular cyclization to yield the desired product.

**Scheme 21:**
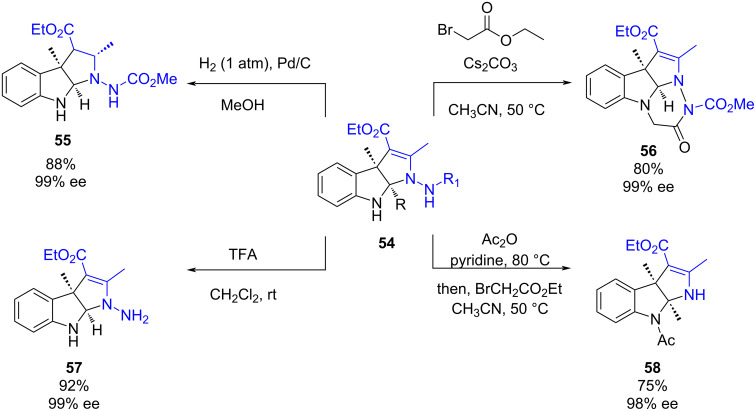
Synthetic applicability of the pyrroloindoline derivatives.

Besides, to show the synthetic applicability of the pyrroloindoline derivatives, various transformations were performed (Scheme 21). Firstly, hydrogenation with palladium on carbon led to the formation of **55** in a good yield. Secondly, an alkylation of the NH of the indole followed by intramolecular cyclization led to tetracyclic derivative **56** in an 80% yield. Next, a deprotection of the azo nitrogen atom led to derivative **57** in a 92% yield. Finally, the N–N bond could be cleaved forming derivative **58** in 75% yield. All the transformations occurred with the retention of the enantioselectivity.

**Scheme 22:**
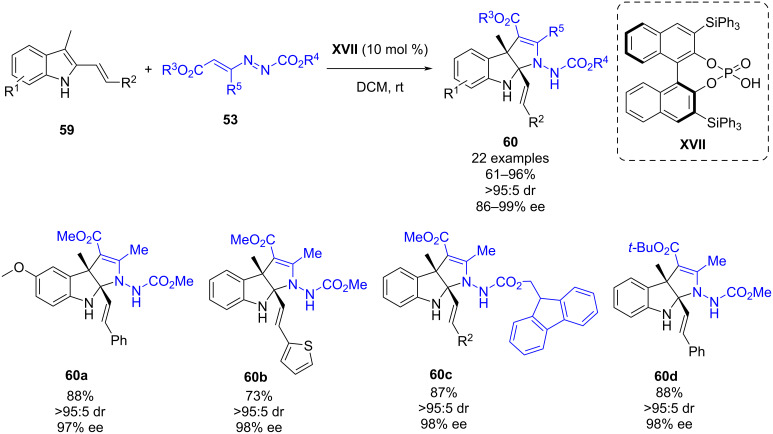
Chiral phosphoric acid-catalyzed (2 + 3) dearomative cycloaddition of 3-alkyl-2-vinylindoles with azoalkenes.

In July 2021, Tian, Wu, Shi and co-workers published a subsequent article of previous work: a chiral phosphoric acid-catalyzed dearomative (2 + 3) cycloaddition of 3-alkyl-2-vinylindoles **59** and azoalkenes **53** (Scheme 22). This methodology afforded chiral pyrroloindolines **60** bearing two tetrasubstituted stereogenic centers in good yields (61–96%) and excellent stereoselectivities (all >95:5 dr, 86–99% ee) by using chiral phosphoric acid **XVII** [44]. The mechanism of the reaction is taking place in an analogous manner as per the previous article, and the chiral phosphoric acid is activating both the azoalkene and the indole through hydrogen bonding. The newly synthesized chiral pyrroloindolines were subjected to biological assays confirming that some of the derivatives exhibit strong anticancer activity against Hela and MCF-7 cell lines.

**Scheme 23:**
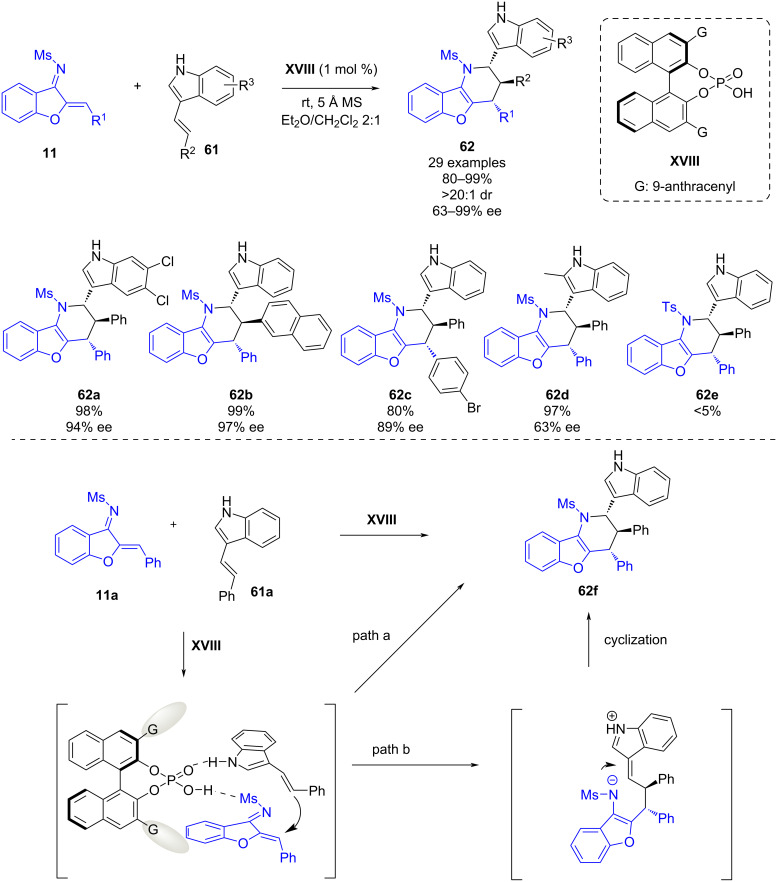
Chiral phosphoric acid-catalyzed asymmetric [4 + 2] cycloaddition of aurone-derived 1-azadienes and 3-vinylindoles.

In early 2021, Mei, Lu and co-worker reported a chiral phosphoric acid-catalyzed asymmetric [4 + 2] cycloaddition of aurone-derived 1-azadienes **11** and 3-vinylindoles **61** (Scheme 23). This methodology enables the synthesis of benzofuran-fused tetrahydropyridines **62** bearing three contiguous stereogenic centers in good yields (80–99%), and excellent diastereoselectivities (>20:1 dr) and good to excellent enantioselectivities (63–99% ee) [45]. In general, good enantioselectivities were obtained with all derivatives, only when 2-methyl-3-vinylindole was tested, a lower enantioselectivity was obtained (**62d**, 63% ee). Additionally, the use of 1-azadiene bearing a tosyl substituent led to traces of cycloaddition product **62e**.

The authors proposed a plausible reaction pathway. Firstly, the chiral phosphoric acid is activating both the azadiene and the 3-vinylindole by hydrogen-bonding interactions. However, from this transition state two possible reaction pathways could explain the observed end product. While in path a, a concerted reaction involving an inverse electron demand aza-Diels–Alder reaction could happen, a stepwise mechanism could be also feasible (path b). In the latter, the addition of the alkene to the azadiene is occurring first and leads to an intermediate which then undergoes an intramolecular cyclization to yield product **62f**. In both pathways, the hydrogen-bonding interaction between the substrate and the product are essential for obtaining the high enantiocontrol in the reaction.

**Scheme 24:**
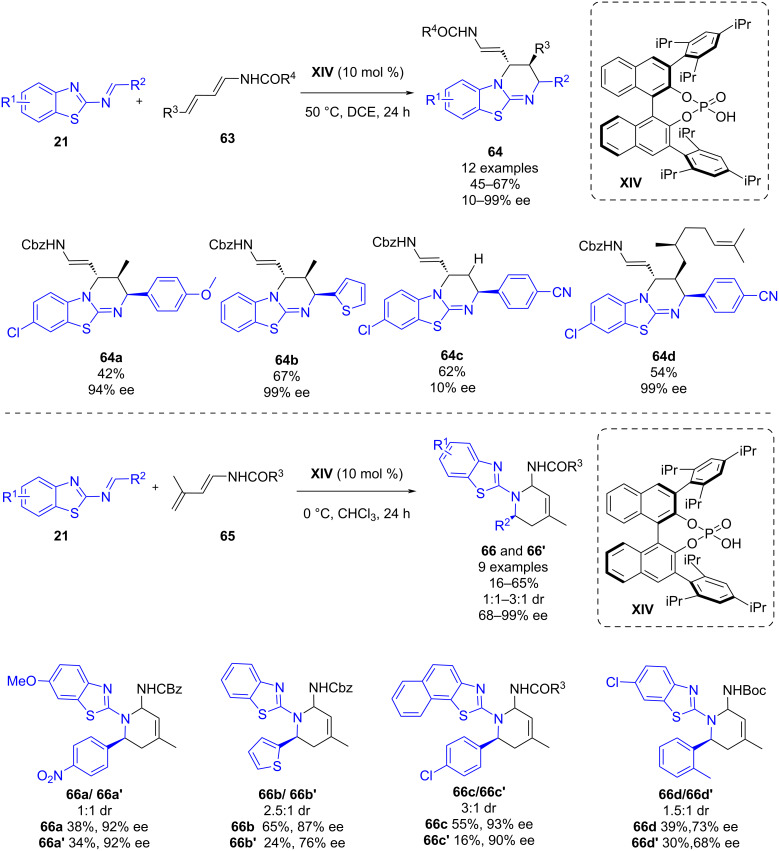
Phosphoric acid-catalyzed enantioselective formal [4 + 2] cycloaddition of dienecarbamates and 2-benzothioazolimines.

In April 2022, Masson and co-workers published a phosphoric acid-catalyzed enantioselective formal [4 + 2] cycloaddition of 2-benzothioazolimines **21** and dienecarbamates **63** and **65** [46]. Firstly, the reaction between 2-benzothioazolimines **21** and 4-substituted dienes **63** was studied, leading to the synthesis of benzothiazolopyrimidines **64** as major product in moderate to good yields (45–67%), complete diastereoselectivities, and in low to high enantioselectivities (10–99% ee) when using organocatalyst **XIV** (Scheme 24). Secondly, the reaction of **21** and 3-substituted dienes **65** led to the production of 1,2,3,4-tetrahydroquinolines diastereomers **66** and **66’** in good to excellent enantioselectivities (68–99% ee) using also chiral phosphoric acid **XIV** (Scheme 24).

**Scheme 25:**
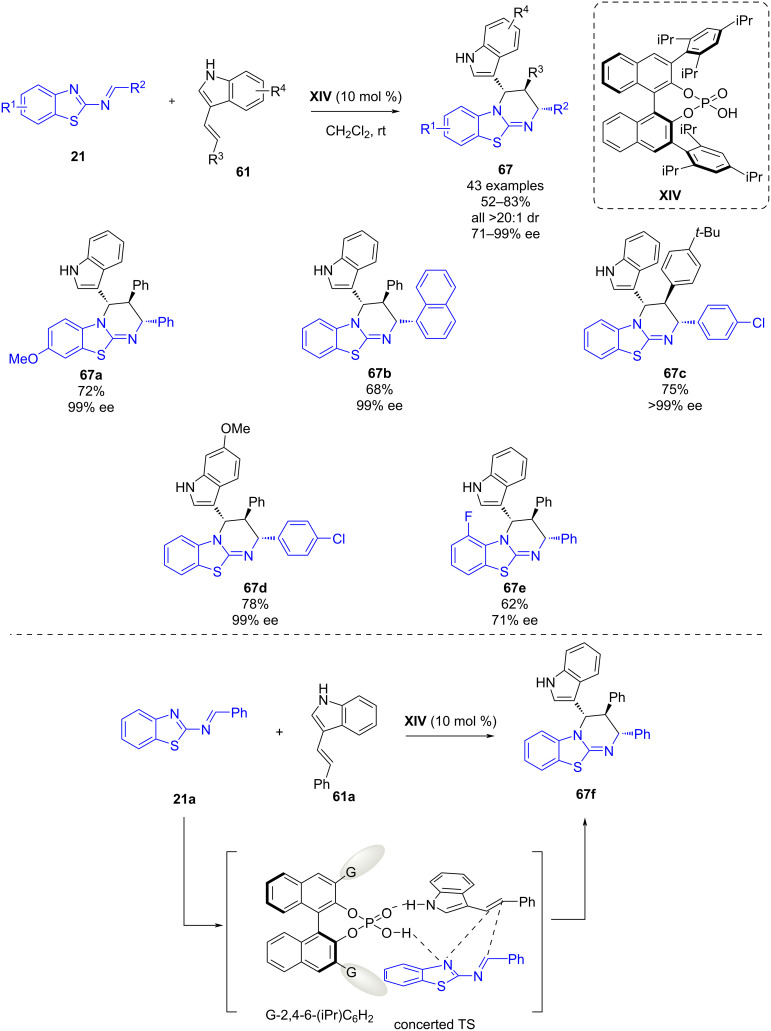
Chiral phosphoric acid-catalyzed asymmetric inverse electron demand aza-Diels–Alder reaction of 1,3-diazadienes and 3-vinylindoles.

In June 2022, Wang, Mei and co-workers reported a chiral phosphoric acid-catalyzed asymmetric inverse electron demand aza-Diels–Alder reaction of 1,3-diazadienes **21** and 3-vinylindoles **61** [47]. After a screening of reaction conditions, chiral phosphoric acid **XIV** was found to be the best organocatalyst, and by using dichloromethane as solvent at room temperature, 43 different benzothiazolopyrimidines **67** were afforded in good yields (52–83%) and good to excellent enantioselectivities (71–99% ee) (Scheme 25). Since the three aromatic residues on the tetrahydropyrimidine ring are placed in a specific *trans*–*trans* relationship, a concerted reaction pathway seems to be occurring. As depicted in Scheme 25, the chiral phosphoric acid acts as a bifunctional catalyst activating both the 1,3-diazadiene and the indole, procuring a transition state in which both reaction partners are approached.

**Scheme 26:**
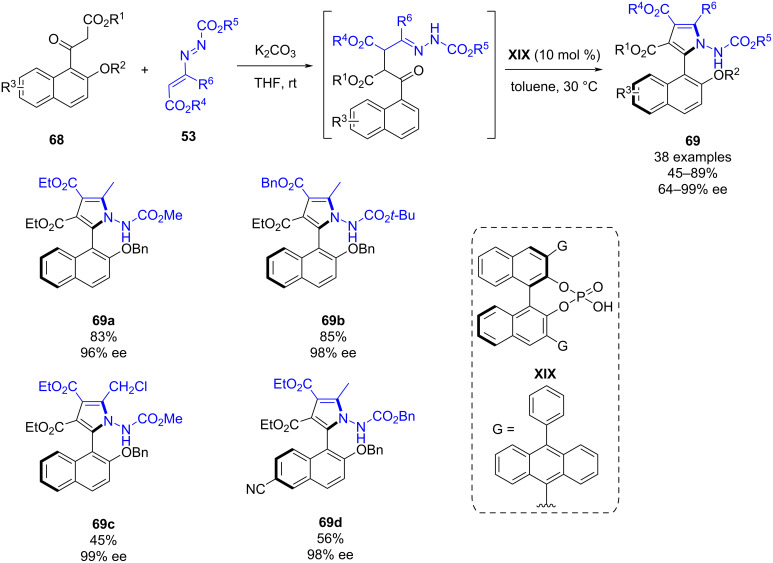
Chiral phosphoric acid-catalyzed asymmetric Attanasi reaction between 1,3-dicarbonyl compounds and azoalkenes.

Also in June 2022, Wang, Xu and Mei reported the chiral phosphoric acid-catalyzed asymmetric Attanasi reaction between 1,3-dicarbonyl compounds **68** and azoalkenes **53** (Scheme 26) [48]. This methodology provides access to *C*_1_-symmetric biarylamino alcohol derivatives (NPNOL) **69** in a wide scope with good yields (45–89%) and good to excellent atroposelectivities (64–99% ee). In order to explain the reaction mechanism and the origin of the enantioselectivity, the authors performed DFT calculations, highlighting the importance of the outer phenyl ring of the chiral phosphoric acid on the enantioinduction.

**Scheme 27:**
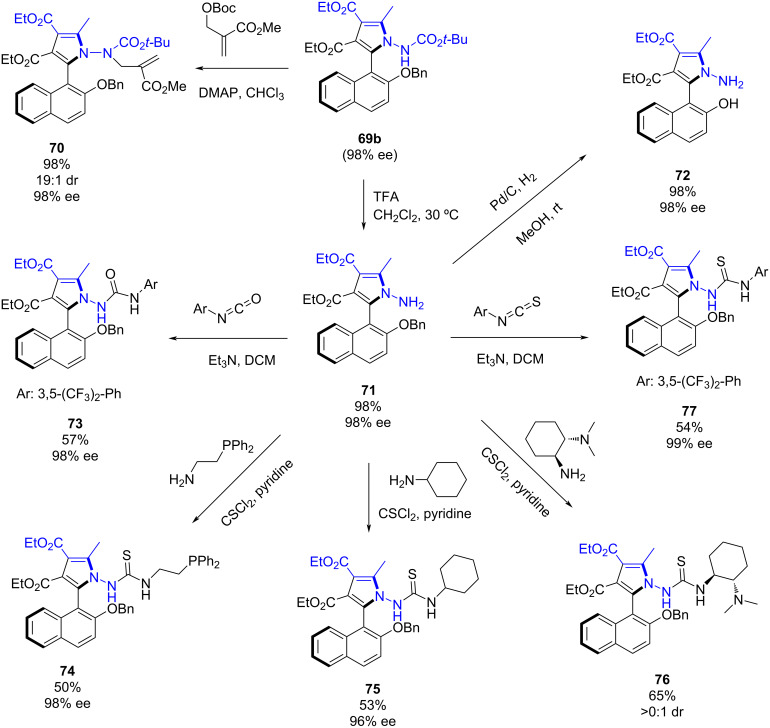
Synthetic applicability of the NPNOL derivatives.

To demonstrate the synthetic applicability of the chiral axial derivatives a variety of transformations were carried out (Scheme 27). An *N-*alkylation of **69b** was performed leading to **70** bearing two stereogenic axes, the biaryl C–C axis and the N–N axis. The removal of the Boc group led to product **71** in a 98% yield. Then, this derivative was subjected to different transformations. Firstly, the hydrogenation using palladium on carbon led to free alcohol derivative **72** in a 98% yield. Next, a urea **73** and various thioureas **74**–**77** were obtained in good yields and with retention of the enantioselectivity, demonstrating the usefulness of the novel methodology for the synthesis of new organocatalysts.

**Scheme 28:**
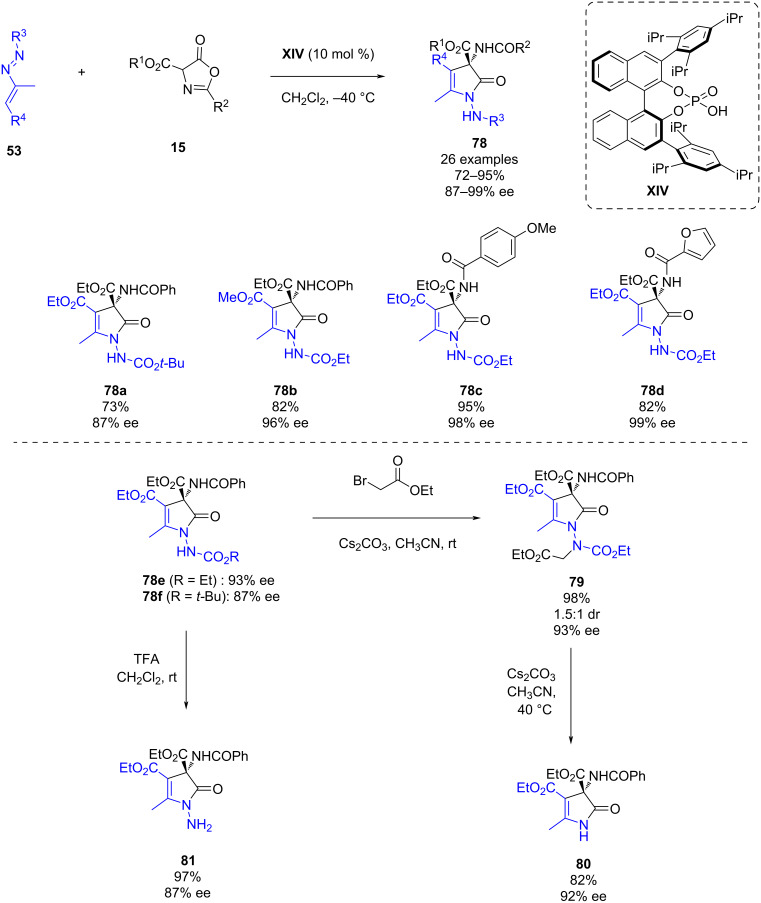
Chiral phosphoric acid-catalyzed asymmetric intermolecular formal (3 + 2) cycloaddition of azoalkenes with azlactones.

In April 2023, Huang, Mei and co-workers reported a chiral phosphoric acid-catalyzed asymmetric intermolecular formal (3 + 2) cycloaddition of azoalkenes **53** with azlactones **15** (Scheme 28). This methodology provides fully substituted 4-pyrrolin-2-ones **78** bearing a quaternary carbon atom in high yields (72–95%) and enantioselectivities (87–99% ee) [49]. Furthermore, to demonstrate the synthetic potential of the methodology, further transformations were carried out. Firstly, the *N-*alkylation of **78e** with ethyl bromoacetate led to the synthesis of tetrasubstituted hydrazine **79** in an excellent yield. This derivative has a newly formed N–N axis conducting to the origin of diastereoselectivity in the reaction. The treatment of **79** with base afforded the pyrrolinone **80** in 82% yield. On the other hand, the deprotection of derivative **78f** led to the hydrazine **81** in an excellent yield. All the derivatizations occurred with the enantiomeric ratio retained.

**Scheme 29:**
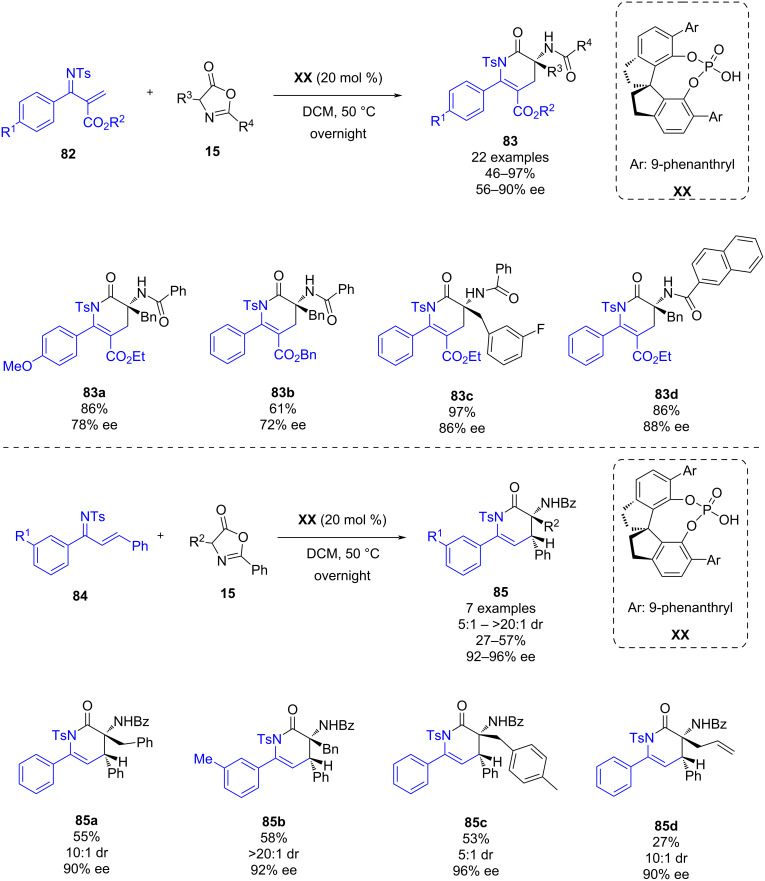
Enantioselective [4 + 2] cyclization of α,β-unsaturated imines and azlactones.

In September 2023, Lan, Huang, Yan and co-workers reported a phosphoric acid-catalyzed enantioselective reaction of acyclic α,β-unsaturated imines and azlactones enabling through an aza-inverse electron demand Diels–Alder reaction, the synthesis of chiral 3**‑**amino-δ-lactams (Scheme 29) [50]. The screening of different Brønsted acids in the reaction between azadiene **82** and azlactone **15** showed that SPINOL-derived catalyst **XX** afforded the best enantioselectivity in the reaction, exploring a scope of 22 derivatives in moderate to excellent yields (46–97%) and enantioselectivities (56–90% ee). Furthermore, under the optimized conditions, the reaction between chalcone-derived azadienes **84** and azlactones **15** was carried out obtaining the chiral 3**‑**amino-δ-lactams **85** in moderate yields (27–57%), good to excellent diastereoselectivities (5:1 to >20:1 dr) and excellent enantioselectivities (92–96% ee).

**Scheme 30:**
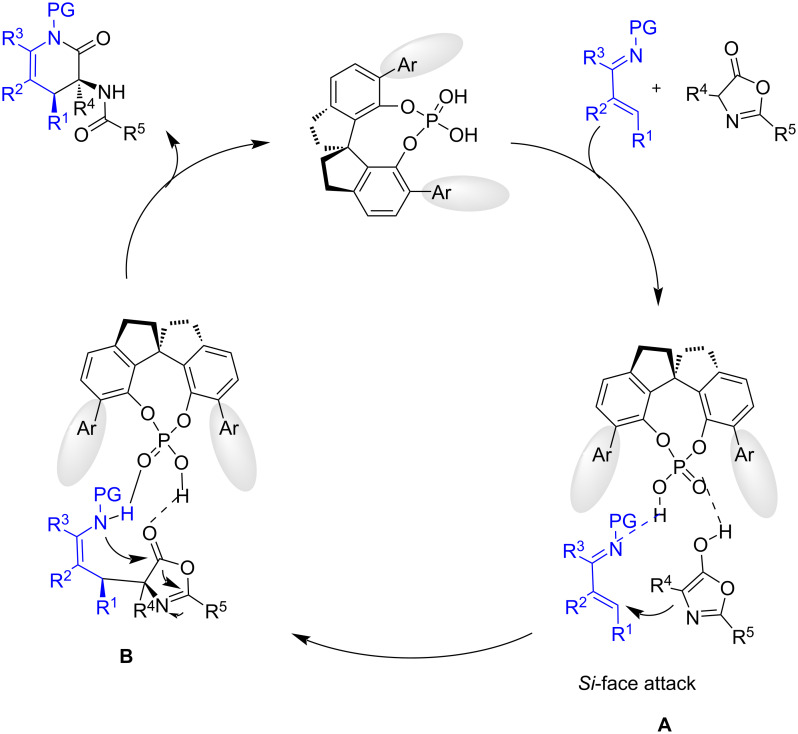
Catalytic cycle for the chiral phosphoric acid-catalyzed enantioselective [4 + 2] cyclization of α,β-unsaturated imines and azlactones.

The proposed reaction pathway is depicted in Scheme 30, the phosphoric acid simultaneously activates the azadiene through hydrogen bonding and the azlactone which is converted to its enol form establishing hydrogen bonding with the phosphoryl group. Next, the 1,4-addition of the azlactone enol through its *Si*-face to the azadiene leads to intermediate **B**, which undergoes an intramolecular lactamization delivering the desired product.

## Conclusion

In this review, an overview of the recent advances of cyclization reactions involving α,β-unsaturated imines catalyzed by non-covalent organocatalysts has been covered. The chance to use 1-azadienes as electron-poor heterodienes together with available electron-rich dienophiles makes the synthesis of a rich variety of useful *N-*heterocyclic derivatives possible. The right selection of the organocatalyst with different reaction partners enables activation providing stereoselective syntheses to access chiral *N-*heterocyclic scaffolds. While those strategies involving hydrogen-bond donors such as bifunctional thioureas and squaramides or chiral Brønsted acids such as chiral phosphoric acids are more established, reports involving cinchona-derived organocatalysts functioning as Brønsted bases remain rarer. Among the different azadienes employed in the different asymmetric transformations, it is common to identify cyclic α,β-unsaturated imines such as aurone-derived azadienes, saccharin-derived azadienes and benzothiazole-derived imines which in combination with different reaction partners such as azlactones, enecarbamates, vinylindoles, 3-isothiocyanatooxindoles, malononitrile, 3-chlorooxindoles or β,γ-unsaturated ketones lead to the synthesis of a great variety of polycyclic *N*-heterocycles.

To summarize, the asymmetric cycloaddition of 1-azadienes is a straightforward methodology which enables the synthesis of structurally distinct *N*-heterocycles, which are difficult to access by other methodologies. Although in recent years a number of studies have been reported, further novel transformations are likely to be reported in the future.

## Data Availability

Data sharing is not applicable as no new data was generated or analyzed in this study.
